# Malaria Vaccines: Current Achievements and Path Forward

**DOI:** 10.3390/vaccines13050542

**Published:** 2025-05-19

**Authors:** Jiayan Chen, Qi Wang, Xiaomeng He, Bei Yang

**Affiliations:** 1 Shanghai Institute for Advanced Immunochemical Studies and School of Life Science and Technology, ShanghaiTech University, Shanghai 201210, China; chenjy22023@shanghaitech.edu.cn (J.C.); wangqi3@shanghaitech.edu.cn (Q.W.); hexm2024@shanghaitech.edu.cn (X.H.); 2 Shanghai Clinical Research and Trial Center, Shanghai 201210, China; 3 Shanghai Frontiers Science Center for Biomacromolecules and Precision Medicine, ShanghaiTech University, Shanghai 200031, China

**Keywords:** malaria vaccines, *Plasmodium*, preclinical, clinical trials, mRNA vaccines, nanoparticle, adjuvants

## Abstract

Malaria remains a significant global health challenge. Although the recent approval of the liver-stage vaccines RTS, S and R21 marks significant progress in malaria control, challenges remain in achieving long-lasting and broad protection. In this review, we provide an overview of the current landscape of malaria control, especially anti-malaria vaccine development. We first review the development of the RTS, S and R21 vaccines, highlighting their efficacy and limitations. We then examine other vaccines in development, including attenuated whole-sporozoite vaccines, as well as blood-stage-targeting vaccines and transmission-blocking vaccines targeting a variety of different immunogens. Additionally, we discuss emerging technologies, such as mRNA-based platforms, nanoparticle delivery systems, and novel adjuvants, assessing their potential to enhance the efficacy and mitigate the waning immunity concerns of most malaria vaccines. We believe that the identification of novel immunogen candidates, together with continued innovation in vaccine design and delivery, will enable us to win the fight against malaria in the future.

## 1. Introduction

Malaria is an infectious disease caused by *Anopheles* mosquito-carried *Plasmodium* parasites. Five species of *Plasmodium* are known to infect humans: *Plasmodium falciparum* (*P. falciparum*), *Plasmodium vivax* (*P. vivax*), *Plasmodium ovale* (*P. ovale*), *Plasmodium malariae* (*P. malariae*), and *Plasmodium knowlesi* (*P. knowlesi*) [1,2]. Of them, *P. falciparum* is the most virulent and is responsible for most malaria-related deaths. The initial symptoms of malaria include high fever, chills, headache, and malaise, which can occur intermittently. Prolonged infections may lead to complications, such as anemia, and can be life-threatening. In 2023, an estimated 263 million confirmed cases were reported, resulting in approximately 600,000 deaths across 83 malaria-endemic countries, primarily in Africa. Notably, 73.7% of these fatal cases occurred in children under five years of age, imposing a significant burden on society [3,4].

To combat malaria, various strategies have been implemented. Preventive measures like indoor residual spraying (IRS) have shown positive effects in preventing malaria infections, with pyrethroids being the primary choice of IRS [5]. Meanwhile, insecticide-treated nets (ITNs) are also effective for malaria prevention [6]. However, resistance to long-lasting insecticides like pyrethroids has developed in many regions [7]. Currently, the WHO specifically recommends pyrethroid–chlorfenapyr-treated nets for areas with pyrethroid resistance [4].

A variety of medicines have also been developed for malaria treatment, including quinolines, artemisinins, and sulfadoxine–pyrimethamine. Nonetheless, due to drug misuse, *Plasmodium* has developed resistance against these medicines to different extents [8,9,10,11,12]. As a result, medicines like chloroquine have been put out of use in many regions. Currently, artemisinin combination therapies (ACTs), which combine artemisinin derivatives with one or more additional drugs, are the first-line treatment against malaria [13]. However, as *Plasmodium* continues to evolve, resistance to artemisinin and its derivatives has also emerged in Africa and Southeast Asia [10].

Vaccines can actively stimulate a protective immune response in vaccinated individuals, thereby offering more direct and sustainable protection compared with IRS and ITNs, especially considering the potential misuse and relatively high replacement cost of the latter. Besides conferring protection against infections, the use of vaccines may also help to break the transmission chain, thereby reducing the spread of malaria and alleviating the healthcare burden and the labor loss. However, efforts to develop malaria vaccines have been marked by multiple setbacks due to both the biological complexity of the parasite and its interaction with the human immune system. Early attempts, such as the synthetic peptide vaccine 66 (SPf66), demonstrated inconsistent efficacy. While initial trials of SPf66 in South America demonstrated modest protection efficacy (28–34%) [14], this protection declined to non-significant levels in African cohorts [15], highlighting the influence of host genetic backgrounds on immune responses. Meanwhile, challenges to vaccine development also come from the parasite’s multi-stage life cycle, each with distinct antigens, thereby making it difficult to elicit broad and lasting immunity [16]. Moreover, the parasite’s high antigenic variability, genetic polymorphisms, and ability to reside within hepatocytes and erythrocytes together shield it from immune surveillance. Consequently, natural immunity to malaria is typically partial, short-lived, and acquired only after repeated infections, complicating the identification of reliable correlates of protection (e.g., effective immunogens) [17]. Furthermore, the absence of robust animal models that accurately recapitulate human malaria infection, and additional barriers, including thermostability, multi-dose regimens, and logistical challenges in resource-limited settings, further hinder vaccine development [16]. Together, these biological and practical obstacles have significantly slowed malaria vaccine development and underscore the necessity of novel approaches to achieve durable, broadly protective immunity.

In past decades, significant efforts have been made in the development of malaria vaccines, with notable successes emerging in recent years. This review highlights the successful development of RTS, S/AS01 and R21/Matrix-M, two recently approved malaria vaccines that have demonstrated efficacy in reducing mortality among children in endemic regions. We will also review other malaria vaccines at various stages of development, including those targeting different stages of the parasite’s lifecycle and employing cutting-edge technologies like mRNA platforms.

## 2. Status of Malaria Vaccines

In recent years, substantial progress has been made in the research and development of malaria vaccines. Scientists have successfully identified a variety of vaccine candidates, several of which have advanced into clinical trials and demonstrated promising preventive effects across diverse populations [18,19,20,21]. These advancements reflect not only an increasing understanding of the malaria parasite and its transmission dynamics but also the effectiveness of novel vaccine technologies.

### 2.1. The Life Cycle of Plasmodium and Corresponding Malaria Vaccines

**Figure 1:**
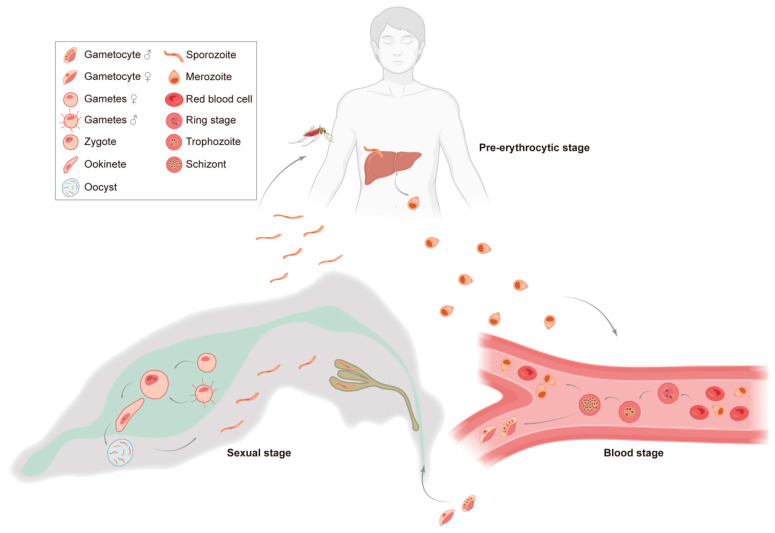
Different stages of the *Plasmodium* life cycle. The life cycle of *Plasmodium* comprises three stages, including the asexual pre-erythrocyte (liver) and erythrocyte stages in humans and the sexual stage in *Anopheles* mosquitoes. The box in the upper left corner depicts different life forms of *Plasmodium* within different hosts.

The complex life cycle of *Plasmodium* can be roughly divided into three stages: the pre-erythrocytic stage (liver stage) and blood stage in humans, and the sexual stage in *Anopheles* mosquitoes. Briefly, infected *Anopheles* mosquitoes transmit *Plasmodium* sporozoites to humans during their blood meals, allowing these sporozoites to invade hepatocytes and develop into thousands of merozoites through a single round of replication (Figure 1) [22]. When the plasma membranes of infected hepatocytes rupture, merozoites are released into the bloodstream, thus initiating the blood stage [23]. During this stage, the asexual merozoites can go through multiple cycles of erythrocyte infections, and 5%~20% of them may differentiate into male and female gametocytes (Figure 1). By chance, gametocytes accumulated in the blood may be picked up by mosquitoes. Within the midgut of the mosquito, gametocytes first develop into male and female gametes and then fertilize each other to form zygotes, which then develop into ookinetes, oocysts, and, finally, sporozoites. These newly produced sporozoites migrate to the salivary glands, where they can be transmitted to the next host through the bite of an *Anopheles* mosquito (Figure 1) [24].

**Figure 2:**
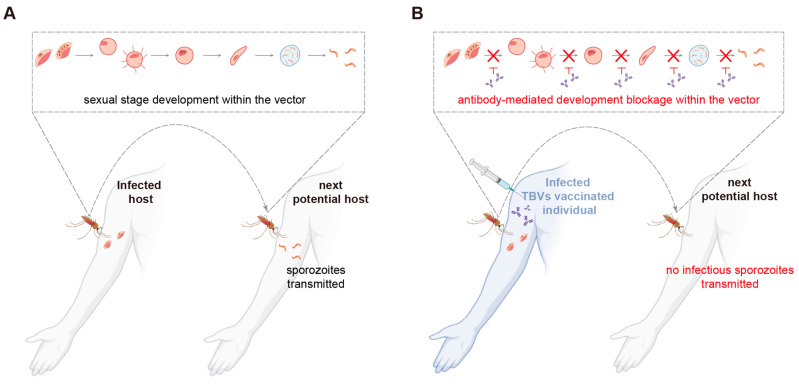
Mechanism of transmission-blocking vaccines. (**A**) The mosquito (vector) intakes gametocytes when feeding on the infected individual (infected host). Within the midgut of the mosquito, these gametocytes develop into gametes, which fuse to form fertilized zygotes. Zygotes then grow into ookinetes that penetrate the midgut epithelium to form oocysts. Within the oocysts, thousands of sporozoites are formed and, later, travel to the salivary glands of the mosquito, where they become ready for transmission to another host during the mosquito’ next blood meal. (**B**) The mosquito intakes both gametocytes and transmission-blocking antibodies when feeding on the infected but TBV-vaccinated individual (infected TBVs vaccinated individual). These transmission-blocking antibodies antagonize the functions of proteins that are essential for the sexual stage development of *Plasmodium*. As a result, no infectious sporozoites are formed and transmitted to another individual during the mosquito’s next blood meal, thereby breaking the transmission cycle of the parasite. Symbols representing different life forms of *Plasmodium* are the same as in Figure 1.

Various vaccines targeting different stages of the *Plasmodium* lifecycle have been designed and tested in the past decades. Among them, vaccines targeting the pre-erythrocytic stage aim to induce immunity against *Plasmodium* sporozoites, thereby preventing them from entering the blood stage [25]. The blood stage is characterized by the asexual reproduction of *Plasmodium* within human erythrocytes, during which infected individuals begin to exhibit clinical symptoms. Vaccines targeting this stage aim to prevent the spread and growth of the parasites and minimize the clinical symptoms associated with malaria [26]. Meanwhile, transmission-blocking vaccines (TBVs) target proteins expressed by *Plasmodium* during the sexual stage, and neutralizing antibodies elicited by TBVs generally work by blocking the parasite’s development in mosquito vectors (Figure 2). Unlike other vaccines, which directly protect humans, the main goal of TBVs is to block the transmission of malaria through herd immunity [27].

### 2.2. WHO-Recommended Malaria Vaccines

**Figure 3:**
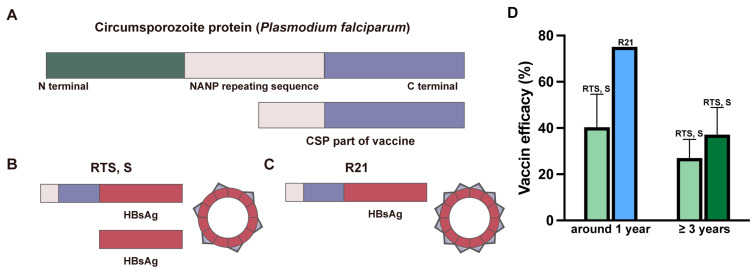
The major components and efficacy of the RTS, S and R21 vaccines. (**A**) Schematic diagrams depicting the domain architecture of the circumsporozoite protein (CSP) of *P. falciparum*, and parts of the CSP that are included in the vaccines. (**B**,**C**) Schematic diagrams depicting the major components of the RTS, S and R21 vaccines. (**D**) Protection efficacy of RTS, S and R21 vaccines at different time points post-vaccination in phase III clinical trials among children from malaria-endemic regions. Light green: 3-dose regimen of RTS, S vaccine; dark green: 4-dose regimen of RTS, S vaccine; light blue: 3-dose regimen of R21 vaccine. Related ClinicalTrials.gov ID: NCT00866619, NCT02207816, and NCT04704830.

Currently, the WHO has approved only two vaccines, RTS, S/AS01 and R21/Matrix-M, for the prevention of *P. falciparum* infections in children in endemic areas. The immunogen of both vaccines is the circumsporozoite protein (CSP) of pre-erythrocytic *Plasmodium* sporozoites (Figure 3A).

RTS, S/AS01: The RTS, S vaccine, marketed under the name of Mosquirix by GlaxoSmithKline, is a virus-like-particle (VLP) vaccine formulated with adjuvant AS01. Its primary immunogen is developed by fusing the C-terminal repeating sequence of the CSP protein from the *P. falciparum* strain NF54 to a 226 amino acid peptide derived from the hepatitis B surface antigen (HBsAg). The fusion proteins, together with additional HBsAgs, then self-assemble into VLPs displaying multiple immunogens on their surfaces (Figure 3B) [28]. The adjuvant AS01, meanwhile, is a liposome-based formulation containing two immunostimulants, i.e., 3-O-deacyl-4′-monophosphoryl lipid A (MPL) and saponin QS-21. Compared with other adjuvants, AS01 elicits a stronger CD4^+^ T-cell response and a higher titer of CSP-specific antibodies [29]. The RTS, S vaccine achieved a 30% protective efficacy among 1~4-year-old children who received three doses of the vaccine [30]. In subsequent phase III clinical trials involving children aged 5–17 months, the vaccine demonstrated a protective efficacy of 53% [31], while in infants aged 6–12 weeks, its efficacy was 30% [32] (Figure 3D). However, the vaccine’s protective efficacy diminished over time. After 38–48 months of follow-up, its protective efficacy in children and infants reduced to 28.3% and 18.3%, respectively [33] (Figure 3D). Moreover, cost-effectiveness analysis of RTS, S, priced at USD 5 per dose for a four-dose regimen, suggests that large-scale implementation could place a significant strain on health budgets in low-resource settings and raise concerns about adherence [34].

R21/Matrix-M: To further improve upon the protective efficacy of the RTS, S vaccine, the R21 vaccine was developed by Oxford University and the Serum Institute of India and utilizes a different adjuvant and contains only the fusion protein but no additional HBsAgs in its formulation (Figure 3C). Compared with the RTS, S vaccine, the R21 vaccine contains a higher proportion of antigenic components derived from the CSP, thereby eliciting a stronger protective immune response with more specific anti-CSP antibodies [35]. The adjuvant Matrix-M included in the R21 vaccine also promotes the early activation of innate immune cells at the injection site and in the draining lymph nodes [36,37]. In a phase II clinical trial with children aged 5–17 months, the R21 vaccine exhibited a protective efficacy of 77%, six months after three doses, and maintained this level for one year [38]. A following phase III clinical trial found a 75% vaccine efficacy after 12 months [39] (Figure 3D).

Compared with other childhood vaccines, no severe safety concerns have emerged during the clinical trials and real-world applications of the RTS, S and R21 vaccines, highlighting their safety [3]. Since 2019, RTS, S has been administered in Ghana, Kenya, and Malawi for children from 5 months old. Thus far, 2 million children living in these malaria-endemic countries have received more than 6 million vaccine doses, which has resulted in a 13% reduction in all-cause mortality and a 22% reduction in severe malaria [3], demonstrating only partial protection. Meanwhile, although clinical trials of R21 have shown promising efficacy, the long-term effectiveness and duration of protection still await evaluation. Additionally, the large-scale implementation of the RTS, S and R21 vaccines faces multiple logistical and financial challenges as both vaccines require a substantial manufacturing capacity and coordinated distribution efforts. At their current prices, securing sustained funding remains critical to support the ongoing production and deployment of both vaccines. Collectively, these constraints pose significant barriers to achieving widespread and equitable vaccine coverage.

### 2.3. Other Vaccines in Development

Despite the approval of the RTS, S and R21 vaccines for children, a vaccine that can provide long-lasting and desired protective efficacy (>75%) across all age groups remains unavailable. Currently, several candidate immunogens are under rigorous investigation, with some progressing into advanced preclinical and clinical trial phases (Table 1).

#### 2.3.1. Whole-Sporozoite Vaccine Candidates

The whole sporozoites of *Plasmodium* during the pre-erythrocytic stage have been a significant focus of vaccine research for many years. However, the use of whole sporozoites in vaccines must consider their pathogenicity. To ensure individuals receiving a *Plasmodium* whole-sporozoite vaccine do not become infected, the sporozoites in whole-sporozoite vaccines are rendered non- or less-pathogenic through radiation, chemoprophylaxis, or genetic attenuation. These attenuation procedures restrict the parasites’ growth and prevent the emergence of blood-stage merozoites that cause disease in humans.

Radiation-attenuated sporozoite vaccines: In 1967, Nussenzweig et al. [53] demonstrated that X-ray-radiated *Plasmodium berghei* sporozoites were partially inactivated and could provoke a protective immune response in mice following immunization. Subsequent experiments involving three volunteers immunized with attenuated sporozoites from the Tamenie strain of *P. falciparum* showed successful protection against autologous sporozoite challenge [54]. In a phase 1 clinical trial, malaria-naive, healthy adult volunteers achieved 77% protective efficacy following three intravenous doses of the radiation-attenuated sporozoite vaccine [55]. However, in a phase 2 clinical trial conducted in infants in a high-transmission malaria setting in western Kenya, no significant protection from *P. falciparum* infection was observed in any dose group [40] (Table 1).

Chemically attenuated sporozoite vaccines: Besides radiation attenuation, chemo-attenuation has also been investigated. Volunteers who received chemo-attenuated sporozoite vaccines were co-administered live sporozoites alongside chloroquine, which inhibited sporozoite development in the body. While chloroquine does not affect sporozoites during the liver stage, it prevents blood-stage parasites from surviving after their release into the circulation [56]. Uninfected volunteers who received three doses of sporozoites combined with chloroquine at four-week intervals achieved 100% protection against controlled human malaria infections (CHMIs) ten weeks after the final dose [41] (Table 1). However, subsequent trials in malaria-infected populations found no significant difference in *Plasmodium* infection rates between the group receiving three doses of the chloroquine-attenuated sporozoite vaccine and the group receiving a placebo [57].

Genetically modified sporozoite vaccines: In 2005, the identification of the *Uis3* (upregulated in infective sporozoite gene 3) gene raised the potential for developing a safe and effective genetically attenuated sporozoite vaccine. Mueller et al. [58] found that *P. berghei* sporozoites lacking the *Uis3* gene were unable to initiate blood-stage infections after invading the hepatocytes of rodents. Depletion of the pre-erythrocytic-stage genes *P52* and *P36* produced a similar early-liver-stage development arrest [59]. In subsequent studies, researchers developed a genetically attenuated sporozoite vaccine, PfGAP3KO, by combining knockouts of *P36*, *P52*, and *SAP1*. Half of the volunteers who received five doses of the PfGAP3KO vaccine were able to resist challenges from autologous *Plasmodium* strains and did not progress to malaria [42] (Table 1).

#### 2.3.2. Blood-Stage Vaccine Candidates

In 1961, researchers discovered that transfusing immunoglobulin G (IgG) from semi-immunized individuals into children reduced parasitemia and alleviated symptoms in the latter [60], thereby directing researchers’ attention toward blood-stage antigens (Table 1). Originally, most investigations focused on merozoite surface protein 1 (MSP1) and apical membrane antigen 1 (AMA1). MSP1 participates in the early attachment and invasion of erythrocytes [61]. When MSP1 is knocked down, parasites cannot be reproduced [62]. Meanwhile, antibodies against MSP1 have also been found to inhibit invasion by *Plasmodium* [63]. Importantly, the immunization of *Aotus* monkeys with MSP1 showed protective immunity against *Plasmodium* challenge [64]. AMA1, meanwhile, is located at the apex of merozoites and mediates the reorientation of merozoites toward erythrocytes [65] by interacting with rhoptry neck protein 2 (RON2) [66], which plays an important role in moving junction formation [67]. Though AMA1 vaccination alone did not induce obvious protection, seven of the eight *Aotus* monkeys vaccinated with the AMA1-RON2L complex were protected against virulent *P. falciparum* infection [68]. However, vaccines targeting these proteins have not demonstrated noticeable protective efficacy in clinical trials [43,44] (Table 1). Later, the discovery of reticulocyte-binding protein homolog 5 (RH5), a conserved protein interacting with the erythrocyte receptor basigin (i.e., CD147) [69], revigorated the blood-stage vaccine field. Moreover, *P. falciparum* cysteine-rich protective antigen (PfCyRPA) is also essential for erythrocyte invasion. RH5, together with parasitic CyRPA and *P. falciparum* RH5-interacting protein (RIPR), assemble into a complex that mediates erythrocyte invasion [70,71,72]. Notably, the immunization of *Aotus* monkeys with RH5 has shown protective effects in CHMI studies [73]. Currently, the RH5.1/AS01_B_ vaccine has gone into phase 1/2a clinical trials. Although it did not fully protect participants, it noticeably reduced the growth rate of *Plasmodium* in vaccinated individuals [46] (Table 1). Meanwhile, in trials involving children from malaria-endemic regions, RH5.1/Matrix-M vaccination elicited an RH5.1-specific response capable of inhibiting ~88% *Plasmodium* growth at a 2.5 mg/mL total IgG concentration [45] (Table 1). In rabbits, the PfCyRPA vaccine induced neutralizing antibodies capable of inhibiting the growth of *P. falciparum* [47] (Table 1). Moreover, neat serum from mice immunized with an mRNA vaccine targeting another blood-stage candidate immunogen, *P. falciparum* glutamic acid-rich protein (PfGARP), manifested >94% growth inhibition activity (GIA) against not only homologous but also heterologous *P. falciparum* strains [48] (Table 1).

#### 2.3.3. Sexual-Stage Vaccine Candidates

To effectively block the transmission of *Plasmodium*, a series of studies have also been directed toward parasitic proteins expressed during the sexual stage (Table 1), including Pfs25, Pfs230, and Pfs45/48. Pfs25 is expressed on the surfaces of the parasite’s zygotes and ookinetes, and monoclonal antibodies against Pfs25 can completely inhibit the development of oocysts in the gut of mosquitoes [74]. Two vaccines targeting Pfs25 are currently under clinical trials, including a VLP vaccine displaying the Pfs25 protein recombinantly purified from plant cells (Pfs25-VLP) and a nanoparticle vaccine with Pfs25 chemically coupled to the carrier protein ExoProtein A (EPA). Nevertheless, after three doses of the Pfs25-VLP vaccine, no significant transmission reduction activity (TRA) was observed in the sera of volunteers across different dosage groups, although ~36.2% TRA was observed for IgG purified from the sera of the 100 ug dose group at 3.75 mg/mL [49] (Table 1). Similarly, clinical studies on Pfs25-EPA demonstrated that the vaccine-induced Pfs25-specific antibody titers decayed rapidly after each shot [50], with standard membrane feeding assays (SMFAs) conducted after the third and fourth doses showing only a weak transmission-blocking effect [50] (Table 1). Meanwhile, Pfs230 and Pfs48/45 form membrane-bound protein complexes on gametocytes, and antibodies against these proteins also exhibit transmission-blocking activities [75]. Participants who received two doses of the Pfs230D1-EPA/Alhydrogel vaccine showed a higher vaccine efficacy than those receiving Pfs25-EPA [76]. Four doses of the Pfs230D1-EPA vaccine provided ~73.7% transmission blocking activity that lasted for 10 weeks [51] (Table 1). Nevertheless, the Pfs48/45-based vaccine-elicited transmission-blocking antibody titers in human serum were too low to amount to significant transmission reduction [52] (Table 1). To summarize, the TBVs currently under study suffer from low immunogenicity and struggle to maintain high antibody titers. Furthermore, TBVs are considered altruistic, as they may not provide direct benefits to vaccinated individuals. Therefore, they may need to be used in combination with other vaccines to achieve malaria elimination.

Taken together, among the various candidate vaccines in development, the blood-stage antigen RH5 and the sexual-stage antigen Pfs230 have demonstrated more promising protective efficacy and favorable safety profiles in preclinical and clinical trials and may warrant prioritization in future malaria vaccine development.

## 3. Potential Use of Advanced Technologies in Malaria Vaccines

Existing malaria vaccines suffer from low immunogenicity and efficacy decay. Hence, there is a pressing need to explore novel vaccine technologies so that a robust immune response can be induced by vaccination with viable immunogens. The evolving landscape of innovative vaccine platforms and adjuvants presents promising avenues for further exploration.

### 3.1. Novel Vaccine Platforms

Through delivering a piece of messenger RNA (mRNA) into the host, mRNA vaccines can instruct the host cells to produce a pathogen-derived protein, thereby triggering a protective immune response against the corresponding pathogen [77]. However, the high cost and logistical challenges of mRNA vaccine deployment remain critical barriers to the global accessibility of this innovative vaccine technology. Most mRNA vaccines require storage at −20 °C to −80 °C, necessitating specialized cold-chain logistics that are 2–3 times more expensive than standard vaccine transport. Despite the high cost and difficulty of distribution, the outstanding efficacy, flexibility, and rapid development of COVID-19 mRNA vaccines highlighted the unprecedented potential of this novel vaccine platform and spurred research to apply this technology to the development of malaria vaccines [78]. Moreover, innovations like lyophilized mRNA vaccines, which retain stability at ambient temperatures, could mitigate cold-chain dependency, although such solutions remain in early-stage trials [79]. PfGARP is a blood-stage immunogen candidate identified through proteomics-based screening [48]. PfGARP is expressed on the exofacial surface of infected erythrocytes and can be recognized by antibodies derived from relatively resistant children. To explore the potential of PfGARP as a vaccine immunogen, nucleoside-modified mRNA encoding PfGARP was encapsulated in lipid nanoparticles (LNPs) and administered to monkeys via intradermal injection across three vaccinations, three weeks apart. This mRNA vaccine successfully elicited an anti-PfGARP humoral response and significantly reduced parasitemia in monkeys challenged with *P. falciparum* [48].

In addition to the mRNA platform, the self-assembling nanoparticle platform offers several advantages that can also enhance the efficacy of vaccines. In nanoparticle vaccines, antigens can be presented on large scaffolds in multiple copies, thus facilitating improved recognition by antigen-presenting cells (APCs) and promoting the activation of helper T cells [80]. Taking the computationally designed I53-50 nanoparticle system as an example, at least 60 antigens can be displayed on the surface of the protein cage assembled from the I53-50A and I53-50B components [81]. In this scenario, the antigens can be presented at a high local density, which, in turn, strengthens the avidity between the immunogen and the B-cell receptors (BCRs) and promotes BCR clustering [82]. Consequently, this promotes a more robust activation of humoral immunity, which is essential for an effective immune response [83,84,85]. This innovative approach may also allow the simultaneous display of multiple antigens, enabling recognition by a diverse range of B-cell receptors [86].

### 3.2. Adjuvant Development and Improvements

Adjuvants are non-specific substances intentionally added to vaccines. The inclusion of adjuvants can enhance or modify the host’s immune response to specific antigens, thereby significantly increasing the vaccine’s efficacy. Traditionally, aluminum adjuvants and water-in-oil adjuvants have been predominantly used in vaccine design. Later on, certain cytokine proteins, such as complement and heat shock proteins, were also discovered to exhibit adjuvant effects. For instance, the fusion of an antigen with complement C3d allows C3d to bind to complement receptor 2 presented on the surfaces of follicular dendritic cells and B cells, thus leading to enhanced antigen presentation and the complete activation of antigen-specific B cells [87]. Meanwhile, Hsp27, a heat shock protein, represents another potent adjuvant that can stimulate both B- and T-cell immune responses [88]. Given their inflammation-stimulating efficacies, toll-like receptor agonists have also been explored as adjuvants. Notably, the use of GLA-SE (a TLR-4 agonist) has significantly improved the immunogenicity and protective effect of the PfCyRPA vaccine [47]. Meanwhile, CpG oligodeoxynucleotides (ODNs) are immune-modulating synthetic oligonucleotides that can stimulate toll-like receptor 9 (TLR9) and trigger Th1 immune responses [89,90,91].

## 4. Conclusions

**Figure 4:**
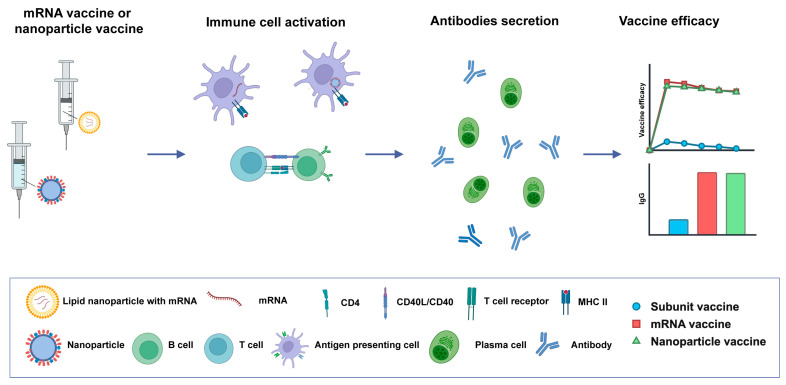
Enhanced immunogenicity of mRNA and nanoparticle-based vaccines. mRNA and nanoparticle vaccines enhance antigen presentation and provide continuous antigen exposure, leading to stronger and longer-lasting immune responses.

Malaria remains a major global public health challenge. Although the development of the RTS, S and R21 vaccines has offered short- to medium-term protection (typically around 1 year) for younger children in high-malaria-burden regions, a vaccine offering full and sustained protection across all age groups is still lacking. To advance malaria vaccine development, continued efforts to identify and optimize novel immunogens, establish reliable correlates of protection, and understand immune responses across diverse genetic backgrounds are essential for guiding rational vaccine design and improving population-wide efficacy. Ideally, a multistage combinatorial vaccine incorporating multiple *Plasmodium* immunogens should be pursued, as simultaneously targeting different phases of the parasite’s life cycle can enhance protective efficacy and elicit broader, more durable immune responses. In parallel, the integration of novel adjuvants and next-generation platforms, such as mRNA and nanoparticle-based technologies, will be critical for boosting immunogenicity and overall vaccine performance (Figure 4). On the implementation front, future research should prioritize thermostable formulations, simplified dosing regimens, and delivery systems that reduce dependence on cold-chain infrastructure. Addressing these scientific and logistical challenges will be key to ensuring sustained, equitable access to malaria vaccines and advancing toward global eradication goals.

Despite ongoing challenges, we believe that with continued scientific innovation and strong support from global health organizations such as the WHO, the development of an effective, long-lasting malaria vaccine remains within reach.

## Figures and Tables

**Table 1:** Current status of malaria vaccines in development.

	Vaccine Immunogen	Adjuvant	Immunogen Type	Current Status	Outcome	Ref.
Pre-erythrocytic stage	PfSPZ	/	Whole sporozoite (radiation attenuation)	Phase 2	No significant protection in infants in endemic regions	[40]
	PfSPZ-Cvac	/	Whole sporozoite (chemical attenuation)	Phase 1	100% homologous CHMI ^1^ efficacy in malaria-naïve healthy adults	[41]
	PfGAP3KO	/	Whole sporozoite (genetic attenuation)	Phase 1	~50% homologous CHMI ^1^ efficacy in malaria-naïve healthy adults	[42]
Blood stage	MSP1	AS02	Subunit	Phase 2	No significant protection in children in endemic regions	[43]
	AMA1	AS01_B_/AS02_A_	Subunit	Phase 1/2a	0% homologous CHMI ^1^ efficacy in malaria-naïve healthy adults	[44]
	RH5.1	Matrix-M	Subunit	Phase 1b	Up to 88% GIA ^2^ of total IgG at 2.5 mg/mL in children in endemic regions	[45]
	RH5.1	AS01_B_	Subunit	Phase 1/2a	Up to 20% PMR ^3^ reduction during homologous CHMI ^1^ in malaria-naïve healthy vaccinated individuals	[46]
	PfCyRPA	GLA-SE	Subunit	Preclinical	~80% GIA ^2^ of total IgG from vaccinated rabbits at 2.5 mg/mL	[47]
	PfGARP	/	mRNA	Preclinical	>94% GIA ^2^ by serum from vaccinated mice	[48]
Sexual stage	Pfs25-VLP	Alhydrogel	Subunit	Phase 1	No TRA ^4^ by neat serum from malaria-naïve healthy adult vaccinated individuals	[49]
	Pfs25-EPA	Alhydrogel	Subunit	Phase 1	9 out of 11 malaria-naïve healthy adult vaccinated individuals displayed >50% TRA ^4^ after 4 doses at 47 ug	[50]
	Pfs230D1-EPA	Alhydrogel	Subunit	Phase 1	73.7% TRA ^4^ 10 weeks post-dose 4 by serum from malaria-experienced Malian adults	[51]
	Pfs48/45	Matrix-M	Subunit	Phase 1	No TRA ^4^ by neat serum from malaria-naïve healthy adult vaccinated individuals	[52]

^1^ Homologous CHMI: controlled human malaria infection with the same parasites as in the vaccine. ^2^ GIA: growth inhibition activity. ^3^ PMR: parasite multiplication rate. ^4^ TRA: transmission-reducing activity.

## Data Availability

Not applicable.
